# Genetics of Arthrogryposis and Macroglossia in Piemontese Cattle Breed

**DOI:** 10.3390/ani10101732

**Published:** 2020-09-24

**Authors:** Liliana Di Stasio, Andrea Albera, Alfredo Pauciullo, Alberto Cesarani, Nicolò P. P. Macciotta, Giustino Gaspa

**Affiliations:** 1 Department of Agricultural, Forest and Food Sciences, University of Torino, Largo Baccini 2, 10095 Grugliasco (TO), Italy; alfredo.pauciullo@unito.it (A.P.); giustino.gaspa@unito.it (G.G.); 2 Associazione Nazionale Allevatori Bovini di Razza Piemontese, strada provinciale Trinita’ 31/A, 12061 Carrù (CN), Italy; andrea.albera@anaborapi.it; 3 Department of Agriculture, University of Sassari, Via De Nicola 9, 07100 Sassari, Italy; acesarani@uniss.it (A.C.); macciott@uniss.it (N.P.P.M.)

**Keywords:** Piemontese breed, arthrogryposis, macroglossia, genetic model

## Abstract

**Simple Summary:**

The study was carried out in order to investigate the genetic background of arthrogryposis and macroglossia in the Piemontese cattle breed, for which limited information is available so far. The genotyping of affected and healthy animals with a high-density chip and the subsequent genome-wide association study did not evidence a single strong association with the two pathologies. Therefore, for arthrogryposis, the results do not support the existence of a single-gene model, as reported for other breeds. Rather, 23 significant markers on different chromosomes were found, associated to arthrogryposis, to macroglossia, or to both pathologies, suggesting a more complex genetic mechanism underlying both diseases in the Piemontese breed. The significant single nucleotide polymorphisms (SNPs) allowed the identification of some genes (*NTN3*, *KCNH1*, *KCNH2*, and *KANK3*) for which a possible role in the pathologies can be hypothesized. The real involvement of these genes needs to be further investigated and validated.

**Abstract:**

Arthrogryposis and macroglossia are congenital pathologies known in several cattle breeds, including Piemontese. As variations in single genes were identified as responsible for arthrogryposis in some breeds, we decided: (i) to test the hypothesis of a similar genetic determinism for arthrogryposis in the Piemontese breed by genotyping affected and healthy animals with a high-density chip and applying genome-wide association study (GWAS), *F*_ST_ and canonical discriminant analysis (CDA) procedures, and (ii) to investigate with the same approach the genetic background of macroglossia, for which no genetic studies exist so far. The study included 125 animals (63 healthy, 30 with arthrogryposis, and 32 with macroglossia). Differently from what reported for other breeds, the analysis did not evidence a single strong association with the two pathologies. Rather, 23 significant markers on different chromosomes were found (7 associated to arthrogryposis, 11 to macroglossia, and 5 to both pathologies), suggesting a multifactorial genetic mechanism underlying both diseases in the Piemontese breed. In the 100-kb interval surrounding the significant SNPs, 20 and 26 genes were identified for arthrogryposis and macroglossia, respectively, with 12 genes in common to both diseases. For some genes (*NTN3*, *KCNH1*, *KCNH2*, and *KANK3*), a possible role in the pathologies can be hypothesized, being involved in processes related to muscular or nervous tissue development. The real involvement of these genes needs to be further investigated and validated.

## 1. Introduction

Arthrogryposis and macroglossia have long been known as congenital abnormalities observed in several cattle breeds [1,2]. Arthrogryposis is characterized by joints contractures with different degrees of severity, which can affect one to four legs, with various associated clinical signs, the most frequent being cleft palate [3]. More than one etiologic event, such as plant toxicosis [4], prenatal viral infections, and a possible hereditary component, have been reported as responsible for the disease occurrence [5]. Less information is available for macroglossia, which consists in the swelling of the tongue that may interfere with the calf’s ability to nurse. The defect is thought to have a genetic basis, but no scientific evidence is available so far.

For both defects, double muscling is considered as a predisposing factor. Already back in the 1963, Lauvergne et al. [6] listed rickets-like troubles and macroglossia among the clinical signs displayed by the hypertrophied animals. The observation that the manipulation of the myostatin gene and, more specifically, the downregulation of its expression resulted in a series of adverse effects, including leg problems and macroglossia, which seems to confirm the negative influence of double muscling [7]. Moreover, macroglossia is one of the primary features of the human Wiedemann-Beckwith syndrome (OMIM 130650), which is clinically similar to muscular hypertrophy in cattle [8].

Both arthrogryposis and macroglossia have been reported for decades in the hypertrophied Piemontese cattle breed. Since the end of 1980s, the National Association of the Piemontese cattle Breeders (ANABORAPI) started to select against these two pathologies by culling Artificial Insemination (AI) bulls with a high percentage of affected progeny. A decrease from 2.74% to 0.34% and from 2.36% to 0.28% in the occurrence of arthrogryposis and macroglossia, respectively, were obtained in the period 1990–2017 (Supplemental Figure S1) as a consequence of this selection strategy (ANABORAPI).

These data seem to support the hypothesis of a genetic background for the defects, but the few investigations in the Piemontese breed did not give conclusive results. Huston et al. [3] suggested that, in the Piemontese, arthrogryposis could be determined by an incompletely penetrant recessive allele, with higher penetrance in males, which seems to be consistent with the ANABORAPI data (Supplemental Figure S1). However, a genome-wide association study carried out on Piemontese calves affected by arthrogryposis and macroglossia genotyped with a medium density (50 K) single nucleotide polymorphism (SNP) BeadChip did not detect clear signals of association for both pathologies [9]. On the contrary, recent studies detected variations in single genes as responsible for arthrogryposis in Angus [10], Swiss Holstein [11], Belgian Blue [12], and Red Danish [13] cattle breeds.

Therefore, the aims of this study were: (i) to test the hypothesis of a similar monogenic determinism for arthrogryposis in the Piemontese cattle breed by genotyping affected and healthy animals with a high-density chip never used in previous studies and applying genome-wide association study (GWAS), *F*_ST_ and canonical discriminant analysis (CDA) procedures, and (ii) to investigate with the same approach the genetic background of macroglossia, for which no information is available so far.

## 2. Materials and Methods

### 2.1. Ethics Statement

No animals were used in the present study. The biological samples belonged to collections available from the ANABORAPI institutional activity. For this reason, the Animal Care and Use Committee approval was not necessary.

### 2.2. Animals, Genotyping, and Data Editing

Animals affected by arthrogryposis or macroglossia were found and sampled by the veterinarians of the ANABORAPI during the routine inspections in the farms registered in the Herd Book of the Piemontese breed. The phenotypic expression of arthrogryposis in the Piemontese breed is very variable, ranging from moderate contracture of the legs to more severe expressions that can be only surgically corrected. As this variability could represent a confounding factor, only animals with extreme expressions of the defect (Supplemental Figure S2a) were considered. Moreover, as in about 3% of the affected animals the two pathologies coexist, only animals affected by a single pathology, arthrogryposis or macroglossia (Supplemental Figure S2), were included in the study, in order to avoid a further confounding effect.

Blood samples were collected from a total of 98 Piemontese male veals: 17 affected by arthrogryposis (Ar), 18 affected by macroglossia (Ma), and 63 healthy (He). The Ar, Ma, and He subjects were distributed in 17, 15, and 59 herds, respectively. All these animals were genotyped with the customized GeneSeek^®^ Genomic Profiler™ Bovine 150 K (Lincoln, NE, USA). As it was difficult to collect a larger number of affected animals for their low incidence due the selection policy, we decided to include 31 additional subjects (16 affected by arthrogryposis and 15 by macroglossia) previously genotyped with the Illumina^®^ BovineSNP50v2 (San Diego, CA, USA). Both 150 K and 50 K markers were mapped on the ARS-UCD1.2 and subjected to quality checks (QC). The QC was performed using PLINK v.1.923 [14] independently for each dataset using the following filters: SNPs with missing rate > 0.02, minor allele frequency (MAF) < 0.05 or deviating from Hardy-Weinberg equilibrium (*p*-values < 10^−6^) were discarded; moreover, only autosomal SNPs with known genomic positions were considered for further analysis. On the subjects’ side, both SNP missingness per individual (>0.02) and individual heterozygosity deviations caused an animal to be removed from the datasets. After data editing, 94 and 31 veals were left in 150 K and 50 K, respectively. Then, the default settings of Beagle software [15] were applied for imputing the 50-K genotypes at 150 K on the whole dataset, obtaining a group of 125 animals (63 He, 30 Ar, and 32 Ma). This dataset was then split into two subsets of 93 (He + Ar) and 95 (He + Ma) individuals each that underwent a new round of QC with the aforementioned settings. A total of 100,791 and 100,907 SNPs were analyzed for the Ar and Ma subsets, respectively.

### 2.3. Statistical Analysis

A genome-wide association study (GWAS) was conducted using two separate case-control designs for the two syndromes following the approaches of [16,17]. The individuals involved in this study were weakly or not related. The genomic relationship matrix off-diagonal elements were, on average, −0.03, −0.03, and −0.02 for Ar, Ma, and He animals, respectively. The negative relatedness values signify that the individuals have genotypes less similar in expectation than the average. The affected and healthy animals were respectively assigned to cases or controls prior to test the allelic associations (–assoc flag in PLINK) performed by a X^2^ test. Genomic-control adjusted *p*-values were plotted against the genomic position for the two analyzed diseases. To tackle the multiple testing issue that arises when thousands of hypotheses are simultaneously tested, we decided to consider as significant the associations with false discovery rate (FDR) [18] below 0.05 using the –adjust flag in PLINK [14]. On the same two datasets, *F*_ST_ analysis was conducted using the Weir and Cockerham [19] estimator implemented in PLINK, and those SNPs exceeding the 99.9th percentile threshold were retained. Then, the *F*_ST_ outliers were merged with SNPs resulting from the GWAS, obtaining two sets of SNPs associated to Ar and Ma, respectively. In order to corroborate the GWAS results, the canonical discriminant analysis (CDA) was run on the top significant SNPs associated to both diseases, jointly considering all the animals and using the CANDISC procedures of SAS software (Sas Institute, Cary, NC, USA). The correlation structure between the top ranked SNPs was used to derive new variables, namely canonical discriminant variables (CAN), able to maximize the separation between predefined groups [20]. If S is the n × p matrix of the p SNP genotypes (coded as 0, 1, or 2 for the “aa”, “Aa”, or “AA” genotypes, respectively) measured in n animals belonging to k groups (three in our cases: Ar, Ma, and He), the ith CAN may be calculated as

CAN_i_ = w_i1_*s*_1_ + w_i2_*s*_2_+⋯+ w_ip_*s*_p_ (1)

where *s_i_* are the centered SNP genotypes and w_ip_ are the raw canonical coefficients for the p analyzed SNP (i.e., the weights of each SNP in the discriminant function). The vectors of coefficients w were obtained with a procedure that involves the eigen-decomposition of a linear transformation of between- and within-group SNP (co)variance matrices [20].

### 2.4. Candidate Gene Detection

Annotated genes were retrieved in the region of 100-kilo base pairs (kb) (50-kb down- and upstream, respectively) surrounding each SNP highlighted by the combined use of GWAS and *F*_ST_. The National Center for Biotechnology Information (NCBI) (http://www.ncbi.nlm.nih.gov) and UCSC Genome Browser Gateway (http://genome.ucsc.edu/) databases were used.

## 3. Results

### 3.1. Genome-wide Association Study and F_ST_

**Figure 1:**
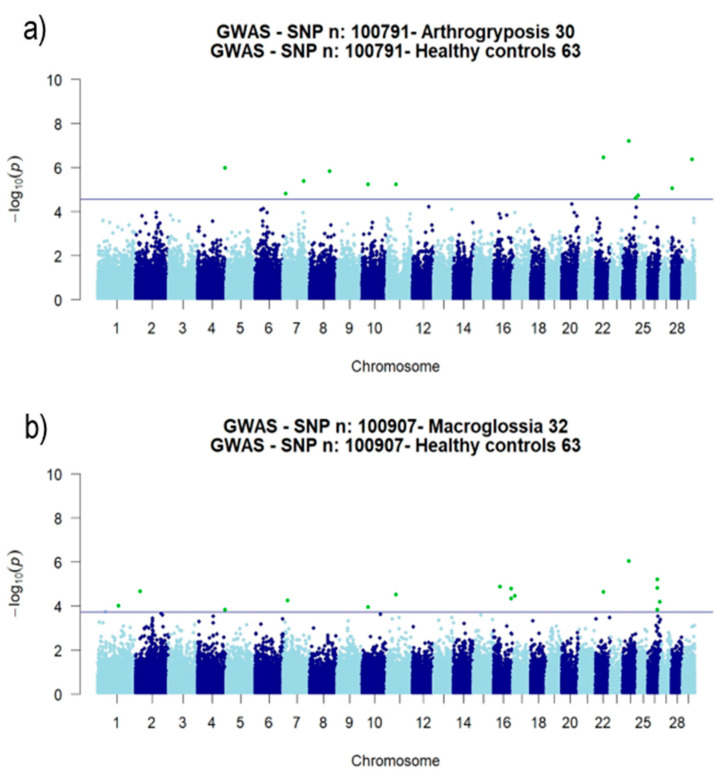
Manhattan plots. (**a**) Healthy vs. arthrogryposis and (**b**) healthy vs. macroglossia. GWAS: genome-wide association study and SNP: single nucleotide polymorphism.

The results of the genome-wide case-control study are shown in Figure 1, which reports the −log10 of genomic-control adjusted *p*-values for the two diseases; in green are highlighted the SNPs that exceeded the chosen threshold. The factor λ was 1.11 and 1.17 for Ar and Ma, respectively, indicating a slight inflation of the statistical test. Quantile-quantile (QQ) plots of the ordered *p*-values for the Ar and Ma case-control GWAS (Supplemental Figure S3) showed that few SNPs strongly deviate from the diagonal identity lines for both diseases.

The *F*_ST_ analysis enabled to highlight 102 and 101 outlier SNPs for Ar and Ma, respectively, (Supplemental Tables S1 and S2) that overcame the 99.9th percentile threshold (Ar vs. He: 0.1401 and Ma vs. He: 0.1425) (Supplemental Figure S4). The combined GWAS and *F*_ST_ approaches were able to identify 23 SNPs that were considered in a further analysis. In particular, seven SNPs were exclusively associated with arthrogryposis (on *Bos taurus* chromosomes, BTAs, 7, 8, 24, 25, 28, and 29) and 11 exclusively with macroglossia (on BTAs 1, 2, 7, 16, 17, and 26), whereas five markers (on BTAs 4, 11, 22, and 24) were in common to both pathologies (Table 1). Consistently, those SNPs presented MAF values markedly deviating in the two groups of animals, as also highlighted by the *F*_ST_ values (from 0.217 to 0.336 and from 0.200 to 0.319 for Ar and Ma, respectively) compared with healthy samples (Supplemental Table S3).

### 3.2. Canonical Discriminant Analysis

The analysis of the squared Mahalanobis distances computed using the top significant SNPs indicated that affected (Ar or Ma) and healthy controls statistically differ (*p* < 0.001) as well as Ar and Ma did within affected animals (Supplemental Table S4).

**Figure 2:**
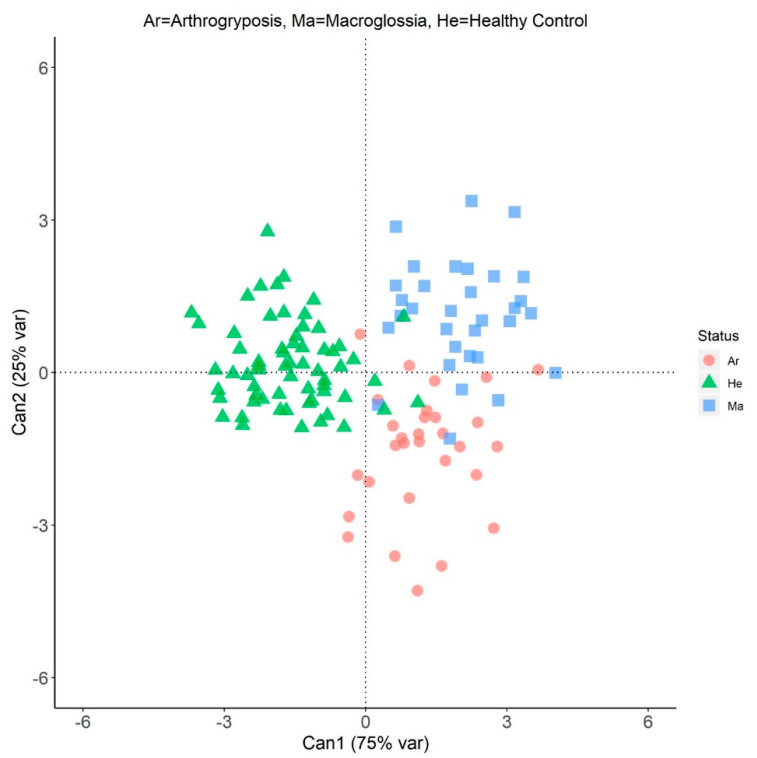
Plot of the individual scores on the two canonical variables (Can1 and Can2) depicted in different colors and symbols according to their health status.

The results of the CDA carried out using the significant markers identified by the GWAS and *F*_ST_ are presented in Figure 2 and Table 1. The two canonical functions explained 75% and 25% of the variance, respectively. When the individual samples were plotted in the new coordinate system defined by the first two discriminant functions, a clear, albeit imperfect, separation between the affected (regardless of the pathology) and the healthy subjects was highlighted (Figure 2). The animals with positive CAN1 scores are mostly the affected animals. In addition, CAN2 allowed to separate the subjects affected by arthrogryposis (with negative scores on CAN2) from those affected by macroglossia (with positive scores), as confirmed by the within-class (Ar, Ma, or He) average scores for CAN1 and CAN2 (Table 1).

The raw canonical coefficients for the identified SNPs had mainly large positive weights for Ma (ranging from −0.09 to 0.72) and negative weights for Ar, even with some exceptions (from −0.57 to 0.62). A rather elusive pattern was ascertained looking at the most deviating canonical weight; conversely, the selected SNPs jointly were able to discriminate between groups of animals. This seems also confirmed looking at the canonical correlation between the SNP genotype and CAN variables (Supplemental Figure S5).

### 3.3. Candidate Gene Detection

The lists of genes that mapped in the interval of 100 kb surrounding the significant SNPs and that may be putatively associated with the diseases are reported in Table 2 and Table 3. As for arthrogryposis, a total of 20 genes were identified in the considered interval, and eight of them included a significant marker, while, for macroglossia, 26 genes were mapped in the highlighted regions, with 10 of them including a marker. Of the identified genes, 12 were in common to both investigated diseases (Table 2 and Table 3).

## 4. Discussion

The present study provides new data on the genetics of arthrogryposis and the first insight into the analysis of macroglossia in the Piemontese breed. An important systematic bias in GWAS often reported in the literature is caused by population stratification due to ethnic/breed admixture and/or close relationships among individuals of case-control studies [21,22]. In our case, a limited inflation of the statistical tests was observed; thus, a genomic-control approach was adopted, since the individuals included in the design belonged to the same breed and were weakly or not related [21].

Interestingly, the combined use of case-control GWAS, *F*_ST_, and CDA highlighted several markers potentially associated with the investigated syndromes. The use of multiple approaches is generally advised in genome-wide analysis [17,23,24]. The use of CDA was recently proposed as an effective tool for improving the discovery rate either alone or in a combination with GWAS, especially when the sample size is reduced [25].

As for arthrogryposis, the results depict a situation different from what was observed in the other investigated breeds [10,11,12,13], where variations in single genes were identified as responsible for the disease. In fact, our data did not evidence a single strong association with the pathology, while they highlighted a number of significant markers located on different chromosomes, suggesting a polygenic mechanism underlying the disease. The joint role of these markers is supported by their ability to separate the three groups of animals according to their health status.

None of the markers for arthrogryposis identified in the Piemontese breed are located within or near the genes reported as causing the disease in the other breeds. In this respect, it is important to underline that also the causal variations found in those breeds were of different types and in different genes: a large deletion encompassing three genes (BTA 16) in Angus [10], a missense mutation in the *MYBPC1* gene (BTA 5) in Swiss Holstein [11], a splicing variant in the *PIGH* gene (BTA 10) in Belgian Blue [12], and a small deletion in the *CHRNB1* gene (BTA 19) in Red Danish [13]. This implies that the genetic determinism of arthrogryposis is not the same in the affected breeds. On the other hand, it must be considered that a large variability in the phenotypic expression of what is called “arthrogryposis” was observed in the breeds studied so far, from lethal consequences, as in Belgian Blue or Angus breeds, to less severe problems, as in the Piemontese. Additionally, at least six types of arthrogryposis with different clinical signs and grades of severity were reviewed by Huston et al. [3] in cattle. Such heterogeneity makes it difficult to clearly define the trait that could explain the differences observed at the genetic level. In all cases, however, the findings of the different studies are compatible with the autosomal recessive mode of inheritance suggested since the earliest studies. The incidences of the two pathologies in the Piemontese breed in the last decades also showed a trend compatible with the case of selection against the recessive phenotype, and this led us to hypothesize the existence of a monogenic determinism similar to what was observed in the other cattle breeds. However, the present data do not support this hypothesis, suggesting that a more complex mechanism is responsible for the disease in the Piemontese breed.

For macroglossia, no previous genetic data exist. The results of the current study highlight a situation comparable to that obtained for arthrogryposis, so that a multifactorial mechanism can be hypothesized also for macroglossia.

The identified SNPs were located within or close to 33 genes, of which 9 and 13 were exclusive for arthrogryposis and macroglossia, respectively, and 11 common to the two pathologies. This is worthy of note, considering that, in the Piemontese breed, both pathologies are sometimes observed in the same animal. Thus, the findings of this study might suggest that the putative candidate genes common to both diseases could be involved in basic physiological processes common to both defects.

In the case of arthrogryposis, seven of the relevant SNPs mapped in coding genes, whereas, for macroglossia, 11 SNPs were located within coding genes. In some cases, it is unclear from the gene annotations their possible involvement in the pathologies. Instead, for other genes, a possible role can be hypothesized, as their products are part of processes related to muscular or nervous tissue developments whose defects are included among the common causes of the pathologies here considered [26].

Among these genes, Netrin3 (*NTN3*) encodes a member (NTN3) of a family of extracellular proteins that act as chemotropic guidance cues for migrating cells and axons during neural development [27]. In mice, it was demonstrated that NTN3 is expressed in muscle cells, and therefore, it may play a role in guiding peripheral axons to their corrected muscle targets [28].

Additionally, *KCNH1* (potassium voltage-gated channel subfamily H member 1) and *KCNH2* (potassium voltage-gated channel subfamily H member 2) genes code for proteins that belong to a complex protein superfamily widely distributed during embryonic development and involved in a wide variety of cell functions. In mice, the two genes are co-expressed in the skeletal muscle during embryogenesis, including the cranial, thoracic, and limb regions [29]. In man, KCNH1 was shown to be involved in myoblast fusion, a complex process that includes withdrawal from the cell cycle, cell-cell interactions, adhesion, alignment, and a final membrane fusion to form the multinucleated skeletal muscle fiber [30].

A possible role can be also suggested for the *KANK3* (KN motif and ankyrin repeat domains 3) gene strongly expressed in different body compartments, including the skeletal muscle, and involved in the control of cytoskeleton formation by negatively regulating actin polymerization [31].

## 5. Conclusions

The overall findings indicate that the genetic determinism of arthrogryposis and macroglossia in the Piemontese breed is more complex than previously believed. In fact, the results do not support the existence of a single-gene model, while suggesting a multifactorial genetic mechanism underlying the investigated pathologies. Several markers significantly associated with both diseases were found, and genes possibly affecting the traits were identified. The real involvement of these genes needs to be further investigated and validated.

## Figures and Tables

**Table 1:** Relevant markers found by the genome-wide association study (GWAS) and *F*_ST_ analysis and raw canonical coefficients (CC) associated to significant single nucleotide polymorphisms (SNPs). Within-class average scores for the two discriminant functions (Can1 and Can2). CC: canonical coefficients.

SNP	GWAS		Raw CC	
	BTA ^1^	Disease ^2^	Can1	Can2
ARS-BFGL-NGS-13673	1	Ma	0.63	0.52
ARS-USDA-AGIL-chr2-17084934-000418	2	Ma	0.27	0.59
ARS-USDA-AGIL-chr4-114395607-000108	4	Ar, Ma	−0.19	0.05
ARS-USDA-AGIL-chr7-12174899-000752	7	Ar	−0.18	−0.14
Hapmap44668-BTA-119022	7	Ar	0.62	−0.26
ARS-USDA-AGIL-chr7-18201332-000761	7	Ma	0.66	0.88
ARS-USDA-AGIL-chr8-84099468-000783	8	Ar	−0.15	−0.72
ARS-USDA-AGIL-chr10-25594159-000176	10	Ar, Ma	−0.37	0.15
ARS-USDA-AGIL-chr11-36809347-000009	11	Ar, Ma	−0.16	0.22
ARS-BFGL-NGS-15423	16	Ma	0.02	0.02
BovineHD1600007856	16	Ma	0.37	0.25
BovineHD4100012725	16	Ma	0.72	−0.11
ARS-USDA-AGIL-chr17-6999864-000318	17	Ma	−0.09	−0.17
ARS-USDA-AGIL-chr22-32285822-000477	22	Ar, Ma	0.19	−0.08
BovineHD2400015279	24	Ar	−0.57	0.56
ARS-USDA-AGIL-chr24-25995108-000530	24	Ar, Ma	−0.17	−0.47
ARS-USDA-AGIL-chr25-1930875-000536	25	Ar	−0.12	0.45
BovineHD4100017966	26	Ma	−0.02	−0.78
BovineHD2600011259	26	Ma	0.31	0.42
BovineHD2600011282	26	Ma	0.42	0.5
BovineHD2600014129	26	Ma	0.59	0.85
BovineHD2800000629	28	Ar	0.56	−1.17
ARS-USDA-AGIL-chr29-39842168-000583	29	Ar	−0.40	−0.65
Within-class average				
Arthrogryposis		Ar	1.23	−1.55
Macroglossia		Ma	2.03	1.13
Healthy control		He	−1.62	0.16

^1^*Bos taurus* chromosome; ^2^ Ar: arthrogryposis and Ma: macroglossia.

**Table 2:** Genes near the SNPs associated to arthrogryposis. The genes including a marker are in bold.

BTA	SNP		Gene		
	Name	Position	Symbol	Name	Location
4	ARS-USDA-AGIL-chr4-114395607-000108	113,596,650	*KCNH2*	Potassium voltage-gated channel, sub-family H, member 2	113,526,185..113,562,025
			*NOS3*	Nitric oxide synthase 3	113,577,075..113,595,527
			***ATG9B***	**Autophagy-related 9B**	113,594,821..113,605,878
			*ABCB8*	ATP binding cassette subfamily B member 8	113,605,775..113,623,178
			*ASIC3*	acid sensing ion channel subunit 3	113,624,088..113,629,085
7	ARS-USDA-AGIL-chr7-12174899-000752	11,085,449	***ZNF333***	**zinc finger protein 333**	11,071,151..11,102,144
			*ADGRE3*	adhesion G protein-coupled receptor E3	11,116,555..11,181,632
	Hapmap44668-BTA-119022	85,227,970	*//*	no genes in the considered interval	
8	ARS-USDA-AGIL-chr8-84099468-000783	82,677,581	***ERCC6L2***	**ERCC excision repair 6 like 2**	82,557,942..82,712,671
10	ARS-USDA-AGIL-chr10-25594159-000176	25,539,231	***OR4E2***	**olfactory receptor, family 4, subfamily E, member 2**	25,539,229..25,540,170
			*OR10G2*	olfactory receptor, family 10, subfamily G, member 2	25,587,007..25,587,963
11	ARS-USDA-AGIL-chr11-36809347-000009	36,957,396	***ACYP2***	**acylphosphatase 2**	36,831,155..37,010,456
			***TSPYL6***	**TSPY like 6**	36,954,224..36,957,858
22	ARS-USDA-AGIL-chr22-32285822-000477	32,169,050	***FRMD4B***	**FERM domain containing 4B**	32,022,767..32,382,939
24	ARS-USDA-AGIL-chr24-25995108-000530	25,684,356	*DSG2*	desmoglein 2	25,602,973..25,653,973
			***DSG3***	**desmoglein 3**	25,666,298..25,699,081
			*DSG4*	desmoglein 4	25,725,711..25,755,364
	BovineHD2400015279	53,288,962	*//*	no genes in the considered interval	
25	ARS-USDA-AGIL-chr25-1930875-000536	1,929,340	*CCNF*	cyclin F	1,947,654..1,965,422
			*TEDC2*	tubulin epsilon and delta complex 2	1,966,625..1,970,947
			*NTN3*	netrin 3	1,975,804..1,978,415
28	BovineHD2800000629	2,661,658	*//*	no genes in the considered interval	
29	ARS-USDA-AGIL-chr29-39842168-000583	39,215,494	*PAG9*	pregnancy-associated glycoprotein 9	39,237,412..39,246,707

**Table 3:** Genes near the SNPs associated to macroglossia. The genes including a marker are in bold.

BTA	SNP		Gene		
	Name	Position	Symbol	Name	Location
1	ARS-BFGL-NGS-13673	86,784,250	*//*	no genes in the considered interval	
2	ARS-USDA-AGIL-chr2-17084934-000418	17,077,000	***ZNF385B***	**zinc finger protein 385B**	16,957,646..17,442,243
			*TRNAC-ACA*	transfer RNA cysteine	17,082,352..17,082,423
4	ARS-USDA-AGIL-chr4-114395607-000108	113,596,650	*KCNH2*	potassium voltage-gated channel, sub-family H, member 2	113,526,185..113,562,025
			*NOS3*	nitric oxide synthase 3	113,577,075..113,595,527
			***ATG9B***	**autophagy related 9B**	113,594,821..113,605,878
			*ABCB8*	ATP binding cassette subfamily B member 8	113,605,775..113,623,178
			*ASIC3*	acid-sensing ion channel subunit 3	113,624,088..113,629,085
7	ARS-USDA-AGIL-chr7-18201332-000761	16,970,401	*CERS4*	ceramide synthase 4	16,897,965..16,933,292
			*CD320*	CD320 molecule	16,952,355..16,957,071
			*NDUFA7*	NADH:ubiquinone oxidoreductase subunit A7	16,960,703..16,967,936
			*RPS28*	ribosomal protein S28	16,968,061..16,969,193
			***KANK3***	**KN motif and ankyrin repeat domains 3**	16,969,360..16,980,814
			*ANGPTL4*	angiopoietin like 4	17,005,585..17,012,655
10	ARS-USDA-AGIL-chr10-25594159-000176	25,539,231	***OR4E2***	**olfactory receptor, family 4, subfamily E, member 2**	25,539,229..25,540,170
			*OR10G2*	olfactory receptor, family 10, subfamily G, member 2	25,587,007..25,587,963
11	ARS-USDA-AGIL-chr11-36809347-000009	36,957,396	***ACYP2***	**acylphosphatase 2**	36,831,155..37,010,456
			***TSPYL6***	**TSPY like 6**	36,954,224..36,957,858
16	BovineHD1600007856	27,494,192	***NVL***	**nuclear valosin-containing protein-like**	27,322,130..27,502,627
			*CNIH4*	cornichon family AMPA receptor auxiliary protein 4	27,529,034..27,543,276
	ARS-BFGL-NGS-15423	72,258,249	***KCNH1***	**potassium voltage-gated channel subfamily H member 1**	72,205,829..72,642,416
	BovineHD4100012725	72,266,300			
17	ARS-USDA-AGIL-chr17-6999864-000318	7,013,884	***LRBA***	**lipopolysaccharide responsive beige-like anchor protein**	6,799,299..7,556,716
22	ARS-USDA-AGIL-chr22-32285822-000477	32,169,050	***FRMD4B***	**FERM domain containing 4B**	32,022,767..32,382,939
24	ARS-USDA-AGIL-chr24-25995108-000530	25,684,356	*DSG2*	desmoglein 2	25,602,973..25,653,973
			***DSG3***	**desmoglein 3**	25,666,298..25,699,081
			*DSG4*	desmoglein 4	25,725,711..25,755,364
26	BovineHD4100017966	40,441,709	***PLPP4***	**phospholipid phosphatase 4**	40,509,729..40,656,785
	BovineHD2600011259	40,460,199			
	BovineHD2600011282	40,517,972			
	BovineHD2600014129	48,680,201	*//*	no genes in the considered interval	

## Supplementary Materials

The following are available online at https://www.mdpi.com/2076-2615/10/10/1732/s1: Figure S1: Trend of arthrogryposis and macroglossia in the Piemontese breed: Incidence from 1990 to 2017. Figure S2: Affected Piemontese veals: (**a**) arthrogryposis and (**b**) macroglossia. Figure S3: Quantile-quantile plot for the case-control genome-wide analysis (in red, the identity line). Figure S4: Distribution of *F*_ST_ values and threshold line of the 99.9th percentile of the ranked *F*_ST_ values for arthrogryposis and macroglossia vs. the healthy control. Figure S5: Canonical correlation between Can1, Can2, and the original SNP results significantly associated with the disease status, represented by different colors. SNPs associated with arthrogryposis (Ar), macroglossia (Ma), or both (Ar_Ma). Table S1: *F*_ST_ outliers (99.9% of ranked *F*_ST_, threshold value: 0.140158) from the arthrogryposis vs. healthy control comparison (n = 102). Table S2: *F*_ST_ outliers (99.9% of ranked *F*_ST_, threshold value: 0.1425518) from the macroglossia vs. healthy control comparison (n = 101). Table S3: Statistics of the significant markers in common between the GWAS and *F*_ST_ analysis. Table S4: Quadratic distance between the animals affected and healthy controls for each comparison.
