# Supplementary material for: Genetics of Arthrogryposis and Macroglossia in Piemontese Cattle Breed
Source: Animals (Basel). 2020 Sep 24;10(10):1732. doi: 10.3390/ani10101732 (PMC7598642; doi:10.3390/ani10101732)
Supplement: Supplementary file 1 [file animals-10-01732-s001.pdf]

Genetics of arthrogryposis and macroglossia in Piemontese cattle breed (Di Stasio *et al*.)

Figure S1. Trend of arthrogryposis and macroglossia in the Piemontese breed: incidence from 1990 to 2017.

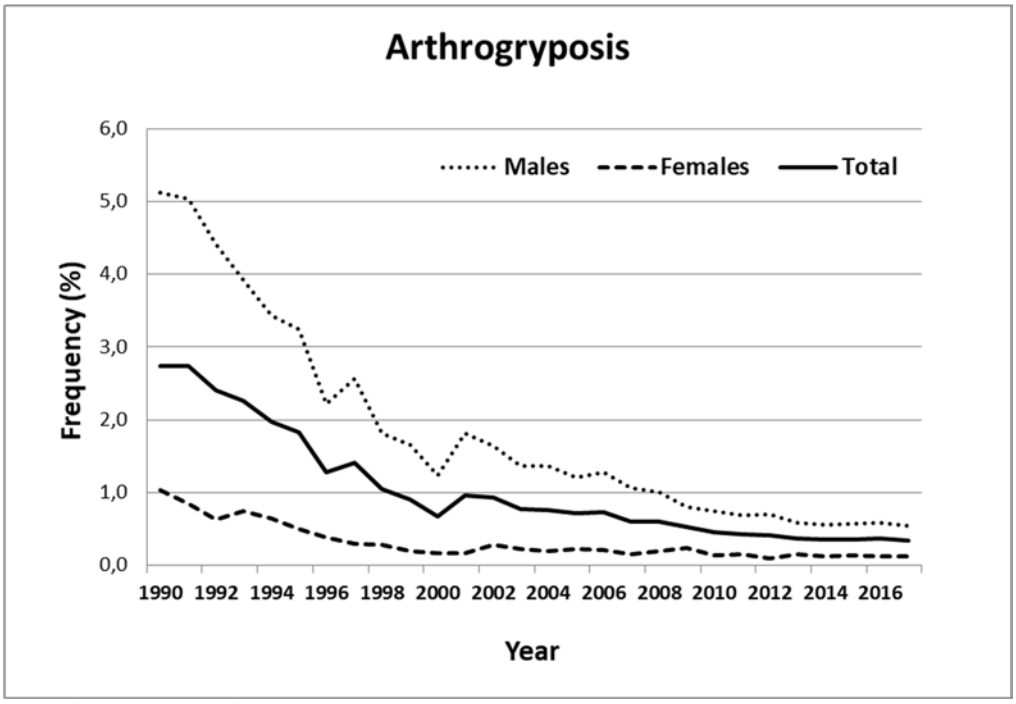

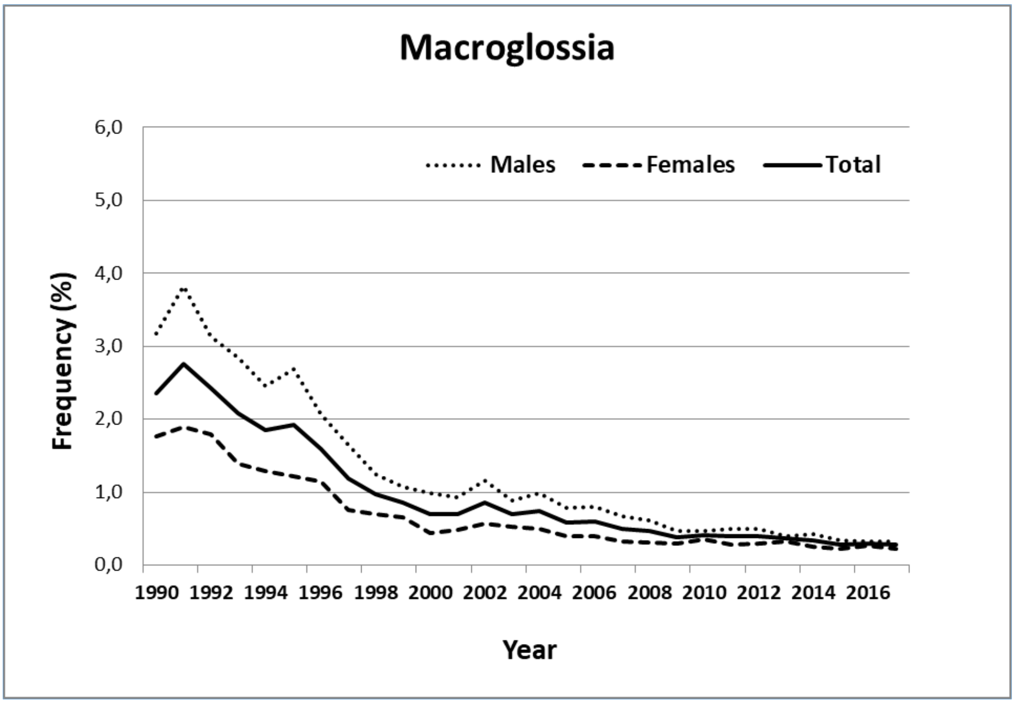

Genetics of arthrogryposis and macroglossia in Piemontese cattle breed (Di Stasio *et al*.)

Figure S2. Affected Piemontese veals: (a) arthogryposis, (b) macroglossia

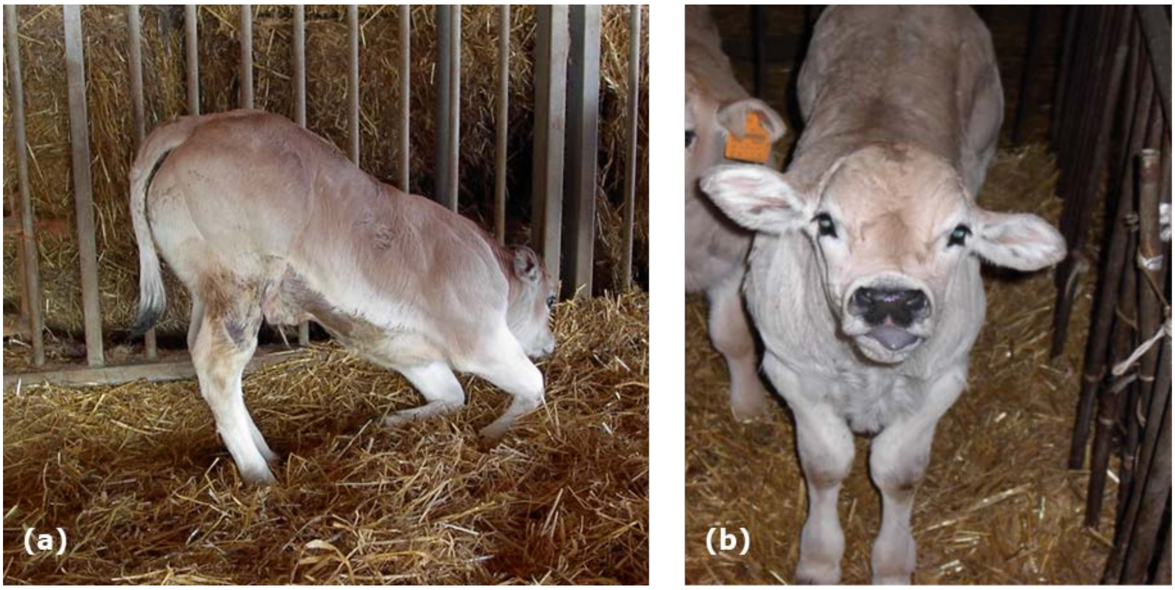

Genetics of arthrogryposis and macroglossia in Piemontese cattle breed (Di Stasio *et al.*)

Figure S3. Quantile-Quantile plot for the case-control genome-wide analysis (in red the identity line).

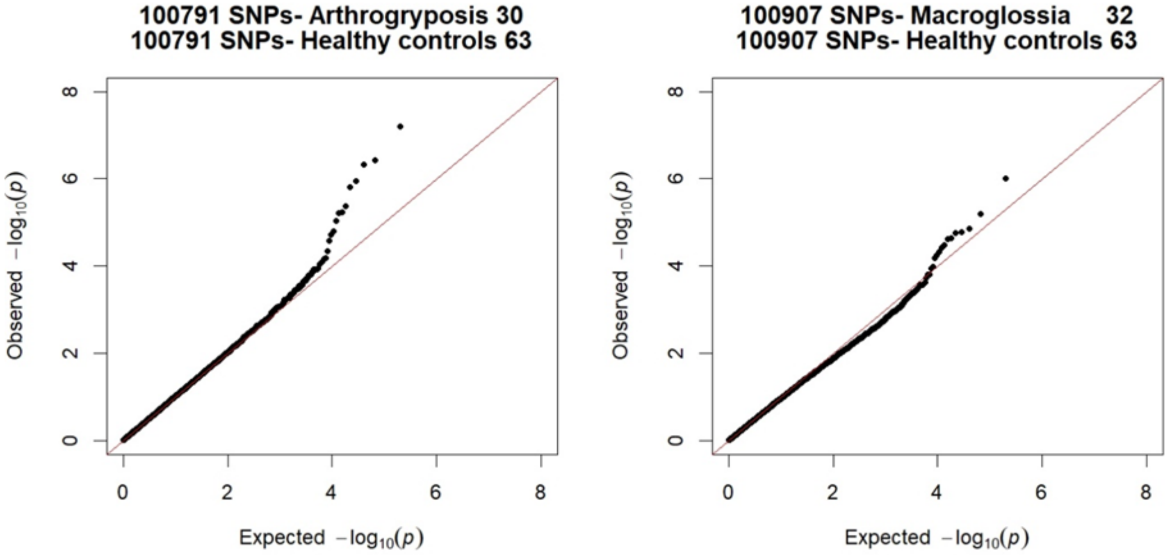

Genetics of arthrogryposis and macroglossia in Piemontese cattle breed (Di Stasio *et al.*)

Figure S4. Distribution of F_{ST} values and threshold line of the 99.9 percentile of the ranked F_{ST} values for Arthogryposis and Macroglossia *vs* Healthy Control.

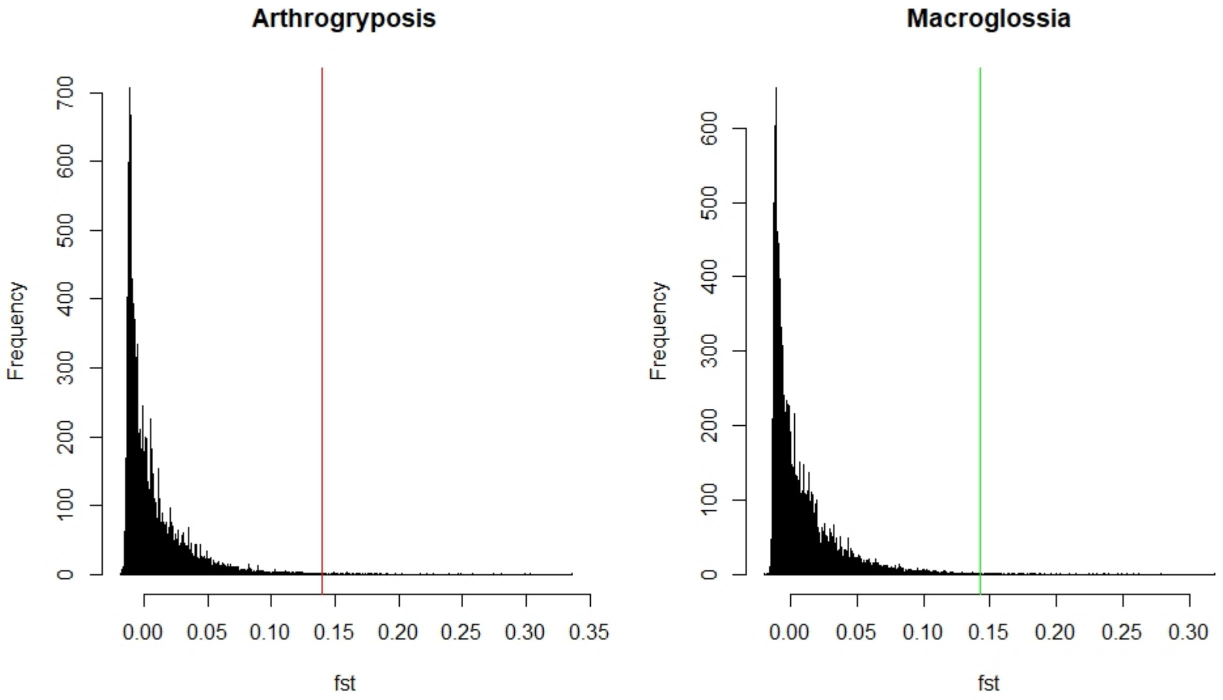

Genetics of arthrogryposis and macroglossia in Piemontese cattle breed (Di Stasio *et al.*)

Figure S5. Canonical Correlation between Can1, Can2 and the original SNPs resulted significantly associated to the disease status, represented by different colors. SNPs associated to Arthrogryposis (Ar), Macroglossia (Ma) or both (Ar_Ma).

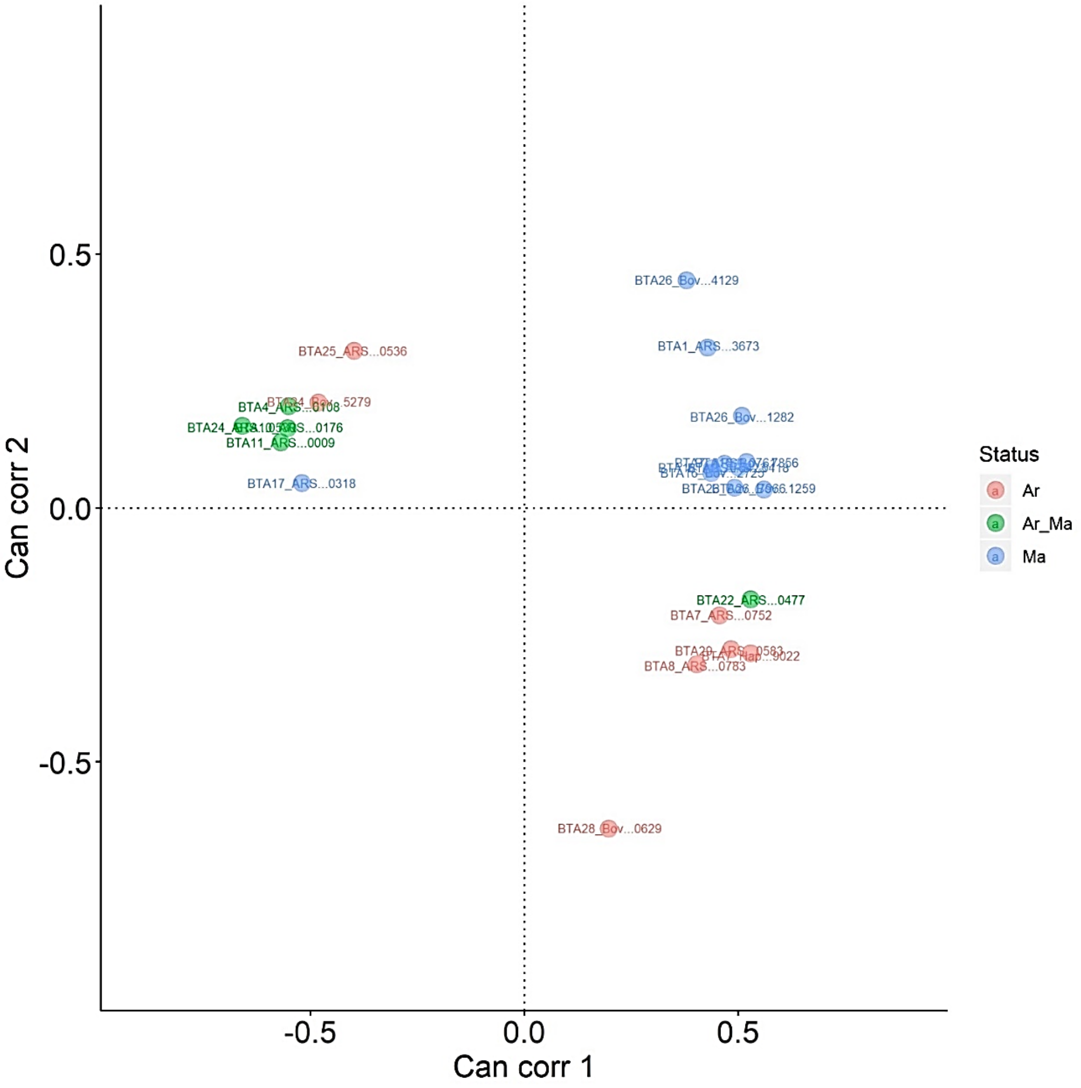

Genetics of arthrogryposis and macroglossia in Piemontese cattle breed (Di Stasio *et al.*)

Table S1. F_{ST} outliers (99.9% of ranked *F*st, Threshold value: 0.140158) for Arthrogryposis *vs* Healthy Control comparison (n=102). The values in boldface are those significantly associated to the disease status in GWAS.

SNP	CHR	BP	FST
ARS-USDA-AGIL-chr1-20798814-000144	1	21300919	0.163455
Hapmap41715-BTA-100026	1	49919784	0.160983
BovineHD0100024372	1	84565987	0.15034
BovineHD4100000493	1	84596584	0.148572
Hapmap60166-rs29019307	1	102616990	0.141233
Hapmap42453-BTA-122047	1	129168140	0.151814
BovineHD0100042448	1	145169000	0.14058
BovineHD0200005190	2	18128284	0.140449
ARS-USDA-AGIL-chr2-25060668-000420	2	25018401	0.176187
BovineHD0200012088	2	41560226	0.160374
BovineHD0200024331	2	84953017	0.184895
BovineHD0200024474	2	85440417	0.173536
BovineHD0200026587	2	92740820	0.156732
BovineHD0200026624	2	92867021	0.149351
BovineHD0200028865	2	100065870	0.158446
ARS-USDA-AGIL-chr3-7904866-000592	3	7907962	0.176345
BovineHD0300006089	3	18919831	0.168184
BovineHD0300013514	3	44065854	0.166102
BovineHD0400001031	4	4079463	0.142244
ARS-BFGL-NGS-55059	4	5545419	0.148487
ARS-BFGL-NGS-6424	4	7225482	0.140158
BovineHD0400017527	4	63635998	0.166591
**ARS-USDA-AGIL-chr4-114395607-000108**	**4**	**113596650**	**0.280511**
Hapmap58253-rs29024365	5	88527468	0.140502
BovineHD0600007417	6	25534440	0.190342
BovineHD0600009523	6	32584576	0.164146
BovineHD0600009530	6	32611538	0.154925
ARS-BFGL-NGS-642	6	34239830	0.190845
Hapmap51534-BTA-103373	6	49974410	0.182351
UA-IFASA-959	6	50802477	0.150394
**ARS-USDA-AGIL-chr7-12174899-000752**	**7**	**11085449**	**0.227596**
BovineHD4100006148	7	64152892	0.143047
BTA-28678-no-rs	7	64375368	0.159007
BovineHD0700019344	7	64378247	0.159007
BTB-01105928	7	64398006	0.159007
**ARS-BFGL-NGS-80444**	**7**	**82683055**	**0.183281**
BovineHD0700025642	7	85132424	0.147645
**Hapmap44668-BTA-119022**	**7**	**85227970**	**0.257593**
BovineHD0700026562	7	88317723	0.147146
Hapmap34237-BES3_Contig337_1046	7	90300540	0.163775
ARS-USDA-AGIL-chr8-84099468-000783	8	82677581	0.274006
BovineHD0800026875	8	88876003	0.155003
BovineHD0900012566	9	44648448	0.159447
**ARS-USDA-AGIL-chr10-25594159-000176**	**10**	**25539231**	**0.24884**
BovineHD1000012199	10	39562997	0.148143
ARS-BFGL-NGS-119395	10	42994125	0.159396
BovineHD1000012994	10	43023306	0.159396

BovineHD1100000551	11	1595594	0.169627
BovineHD1100000556	11	1601521	0.154424
BovineHD1100000561	11	1606505	0.154424
ARS-BFGL-NGS-41117	11	6693294	0.148833
BovineHD1100002555	11	6738342	0.16708
BTB-00461926	11	16242950	0.144661
**ARS-USDA-AGIL-chr11-36809347-000009**	**11**	**36957396**	**0.246966**
Hapmap46918-BTA-105873	11	83570038	0.151757
BovineHD1100027823	11	95693625	0.171799
ARS-BFGL-NGS-5179	11	97906954	0.178678
ARS-BFGL-NGS-113310	11	98633647	0.141385
BTB-01592095	12	66498456	0.197341
BovineHD1200024201	12	79674535	0.154692
BovineHD1200025058	12	82379113	0.142208
ARS-USDA-AGIL-chr13-75913474-000249	13	75201557	0.190677
BovineHD1400023269	14	79983698	0.158062
BovineHD1500008537	15	31081968	0.141333
BovineHD1600006434	16	22370308	0.140784
BovineHD1600006588	16	23086489	0.181091
BovineHD1600008113	16	28191223	0.173707
ARS-USDA-AGIL-chr16-56117438-000313	16	54660223	0.17656
ARS-USDA-AGIL-chr17-6999864-000318	17	7013884	0.18112
BovineHD1700007933	17	27486221	0.148445
Hapmap34511-BES2_Contig398_735	17	46836856	0.142158
ARS-USDA-AGIL-chr17-74081633-000036	17	72089995	0.154692
BovineHD1800002506	18	7129817	0.140158
BTA-08388-no-rs	19	55502869	0.141078
BovineHD1900015846	19	55506254	0.141078
ARS-BFGL-NGS-69971	20	43159533	0.202495
BTB-00788635	20	53721563	0.185644
ARS-BFGL-NGS-111615	20	61971945	0.149399
BovineHD2000017478	20	61980304	0.149399
BovineHD2000017481	20	61992013	0.178033
BovineHD4100014890	20	68194875	0.148907
BovineHD2200002328	22	7706294	0.168608
BovineHD2200004208	22	14329021	0.14866
BovineHD2200004379	22	14363195	0.14866
BovineHD2200004382	22	14365532	0.14866
BovineHD2200004615	22	15305029	0.15234
BovineHD2200005234	22	18212564	0.160429
**ARS-USDA-AGIL-chr22-32285822-000477**	**22**	**32169050**	**0.302963**
BovineHD2300013637	23	47140220	0.142462
BovineHD2300014310	23	49251604	0.143201
**ARS-USDA-AGIL-chr24-25995108-000530**	**24**	**25684356**	**0.335929**
BovineHD2400012922	24	46208577	0.146954
**BovineHD2400015248**	**24**	**53199984**	**0.174055**
BovineHD2400015279	24	53288962	0.216671
BTA-58706-no-rs	24	56368404	0.197316
**ARS-USDA-AGIL-chr25-1930875-000536**	**25**	**1929340**	**0.222049**
BovineHD2500008188	25	29223697	0.16096
BovineHD2600011259	26	40460199	0.151322
**BovineHD2800000629**	**28**	**2661658**	**0.239498**
**ARS-USDA-AGIL-chr29-39842168-000583**	**29**	**39215494**	**0.299438**
ARS-BFGL-NGS-89746	29	48833336	0.169848
BovineHD2900014578	29	48869098	0.15995

Genetics of arthrogryposis and macroglossia in Piemontese cattle breed (Di Stasio *et al.*)

Table S2. F_{ST} outliers (99.9% of ranked F_{ST}, Threshold value: 0.1425518) from Macroglossia *vs* Healthy Control comparison (n=101). The values in boldface are those significantly associated to the disease status in GWAS.

SNP	CHR	BP	FST
BovineHD0100002309	1	7654936	0.171235
ARS-USDA-AGIL-chr1-20798814-000144	1	21300919	0.167485
BovineHD0100009188	1	31896161	0.195778
**ARS-BFGL-NGS-13673**	**1**	**86784250**	**0.213922**
BovineHD0100028911	1	100485690	0.147867
BTA-109427-no-rs	1	108225670	0.160244
BovineHD0100046611	1	134961350	0.155866
**ARS-USDA-AGIL-chr2-17084934-000418**	**2**	**17077000**	**0.248413**
BovineHD0200010478	2	35606140	0.151773
BTB-01246228	2	35622796	0.151773
BTB-00295753	2	35722982	0.153158
ARS-BFGL-NGS-18591	2	44081015	0.157688
BTB-00183404	2	54608036	0.15455
BovineHD0200019365	2	66617723	0.158714
BovineHD0200019662	2	67729511	0.165908
BovineHD0200019919	2	68638010	0.160298
BovineHD0200019935	2	68711981	0.181503
BovineHD0200019945	2	68757260	0.148421
ARS-BFGL-NGS-105016	2	69929788	0.172882
BovineHD0200020580	2	71325436	0.143988
BovineHD0200030786	2	106185590	0.190708
BovineHD0200032009	2	110303270	0.187975
BovineHD0200032928	2	113239520	0.156383
BovineHD0300011574	3	37209678	0.156139
BovineHD0300014636	3	48006297	0.146695
BovineHD0300016810	3	55490893	0.145606
BTB-00156546	3	110260420	0.163243
BovineHD0400001564	4	5558293	0.172678
ARS-USDA-AGIL-chr4-66003777-000640	4	65605611	0.183037
BovineHD0400018125	4	65649876	0.166029
**ARS-USDA-AGIL-chr4-114395607-000108**	**4**	**113596650**	**0.199883**
ARS-USDA-AGIL-chr5-58067406-000679	5	57741083	0.155274
BovineHD0500024333	5	85511462	0.163243
BovineHD0600007417	6	25534440	0.164243
BovineHD0600034042	6	114186020	0.178356
BovineHD0700032903	7	8466147	0.167743
ARS-USDA-AGIL-chr7-12174899-000752	7	11085449	0.145395
ARS-USDA-AGIL-chr7-18201332-000761	7	16970401	0.227314
ARS-BFGL-NGS-76969	7	22273701	0.150336
BTB-00300946	7	22318140	0.150336
BovineHD0800004691	8	15068593	0.152569
BovineHD0900012413	9	44165518	0.14631
BovineHD0900012426	9	44210242	0.152872
BovineHD0900013157	9	47142867	0.17341
**ARS-USDA-AGIL-chr10-25594159-000176**	**10**	**25539231**	**0.209432**
ARS-BFGL-NGS-3998	10	76862396	0.190779
BovineHD1100006675	11	22104302	0.178071

**ARS-USDA-AGIL-chr11-36809347-000009**	**11**	**36957396**	**0.238806**
BovineHD1100014525	11	49639983	0.150463
UA-IFASA-8854	11	49640162	0.180605
BovineHD1200001210	12	4031682	0.15694
BovineHD1300006670	13	22520015	0.174371
BovineHD1300009628	13	32729625	0.151251
ARS-USDA-AGIL-chr13-75913474-000249	13	75201557	0.147333
ARS-BFGL-NGS-23787	13	75318964	0.183593
BovineHD1300024267	13	82633098	0.168178
BovineHD1400012961	14	43721075	0.166297
BovineHD1500002753	15	10356099	0.143747
BovineHD1500003530	15	14060730	0.147771
BovineHD1500005214	15	20276441	0.149421
Hapmap41518-BTA-36331	15	31693526	0.187738
Hapmap47270-BTA-36334	15	31736591	0.187738
BovineHD1500024160	15	81377169	0.175028
BovineHD1600000186	16	1003557	0.146727
**BovineHD1600007856**	**16**	**27494192**	**0.261461**
ARS-USDA-AGIL-chr16-56117438-000313	16	54660223	0.157241
**ARS-BFGL-NGS-15423**	**16**	**72258249**	**0.252403**
**BovineHD4100012725**	**16**	**72266300**	**0.229767**
**ARS-USDA-AGIL-chr17-6999864-000318**	**17**	**7013884**	**0.235469**
ARS-BFGL-NGS-103372	18	8716520	0.174845
BovineHD1900005248	19	17986878	0.145586
BovineHD1900006972	19	23611776	0.144014
ARS-BFGL-BAC-31839	19	24200990	0.153827
BovineHD1900007478	19	24895443	0.146071
BovineHD1900011893	19	41082405	0.158346
BovineHD1900014271	19	50510410	0.145452
ARS-BFGL-NGS-102633	20	65154115	0.142608
BovineHD2100000329	21	2457572	0.148012
BovineHD2100000332	21	2498610	0.148012
BovineHD2100007679	21	26142023	0.151512
BovineHD2100019350	21	64347656	0.145413
BovineHD2200000496	22	1893009	0.179981
**ARS-USDA-AGIL-chr22-32285822-000477**	**22**	**32169050**	**0.245891**
ARS-USDA-AGIL-chr22-57797674-000484	22	57141089	0.180697
**ARS-USDA-AGIL-chr24-25995108-000530**	**24**	**25684356**	**0.318801**
BovineHD2500008188	25	29223697	0.150009
BovineHD2500008631	25	31063416	0.151453
BovineHD2500010631	25	37577205	0.143663
ARS-BFGL-NGS-63916	26	34158439	0.160509
ARS-BFGL-NGS-43058	26	37139478	0.143914
**BovineHD4100017966**	**26**	**40441709**	**0.202795**
**BovineHD2600011259**	**26**	**40460199**	**0.278343**
**BovineHD2600011282**	**26**	**40517972**	**0.257321**
BovineHD2600011355	26	40772885	0.187073
BovineHD2600011508	26	41287739	0.15389
Hapmap39279-BTA-61630	26	42800673	0.154816
BovineHD2600013896	26	47886423	0.172018
**BovineHD2600014129**	**26**	**48680201**	**0.224353**
BovineHD2600005868	26	51261037	0.178441
ARS-USDA-AGIL-chr29-39842168-000583	29	39215494	0.154244
BovineHD2900014776	29	49812895	0.160252

Table S3. Statistics of the significant markers in common between GWAS and F_{ST} analysis

Pathology	SNP	BP	BTA	N	A1	A2	Pvalue HWE	MAF	MAF Affected	MAF Unaffected	Nominal p-values	GC-pvalue	FDR (BH)	FST
Arthrogryposis	**ARS-USDA-AGIL-chr4-114395607-000108***	113596650	4	93	A	G	7.69E-05	0.29	0.03	0.41	9.91E-08	1.12E-06	2.50E-03	0.281
Arthrogryposis	ARS-USDA-AGIL-chr7-12174899-000752	11085449	7	93	A	G	4.06E-02	0.24	0.45	0.13	2.28E-06	1.56E-05	2.30E-02	0.228
Arthrogryposis	Hapmap44668-BTA-119022	85227970	7	93	A	G	8.22E-01	0.36	0.62	0.24	4.97E-07	4.34E-06	8.35E-03	0.258
Arthrogryposis	ARS-USDA-AGIL-chr8-84099468-000783	82677581	8	93	A	G	1.83E-03	0.14	0.33	0.05	1.50E-07	1.58E-06	3.02E-03	0.274
Arthrogryposis	**ARS-USDA-AGIL-chr10-25594159-000176***	25539231	10	93	G	A	2.12E-01	0.46	0.20	0.59	7.33E-07	6.01E-06	9.24E-03	0.249
Arthrogryposis	**ARS-USDA-AGIL-chr11-36809347-000009***	36957396	11	93	A	C	1.68E-03	0.37	0.12	0.49	7.26E-07	5.96E-06	9.24E-03	0.247
Arthrogryposis	**ARS-USDA-AGIL-chr22-32285822-000477***	32169050	22	93	A	G	6.27E-04	0.26	0.52	0.13	2.67E-08	3.71E-07	1.17E-03	0.303
Arthrogryposis	**ARS-USDA-AGIL-chr24-25995108-000530***	25684356	24	93	A	C	1.23E-05	0.31	0.02	0.44	3.31E-09	6.44E-08	3.33E-04	0.336
Arthrogryposis	BovineHD2400015279	53288962	24	93	G	A	1.46E-01	0.49	0.73	0.37	4.32E-06	2.67E-05	3.63E-02	0.217
Arthrogryposis	ARS-USDA-AGIL-chr25-1930875-000536	1929340	25	93	G	A	4.44E-03	0.39	0.15	0.51	2.96E-06	1.95E-05	2.72E-02	0.222
Arthrogryposis	BovineHD2800000629	2661658	28	93	C	A	1.34E-01	0.17	0.37	0.08	1.22E-06	9.20E-06	1.36E-02	0.239
Arthrogryposis	ARS-USDA-AGIL-chr29-39842168-000583	39215494	29	93	A	T	6.74E-03	0.31	0.58	0.18	3.47E-08	4.63E-07	1.17E-03	0.299
Macroglossia	ARS-BFGL-NGS-13673	86784250	1	95	A	C	1.00E+00	0.11	0.25	0.03	3.60E-06	1.05E-04	2.80E-02	0.214
Macroglossia	ARS-USDA-AGIL-chr2-17084934-000418	17077000	2	95	A	G	3.94E-02	0.45	0.70	0.32	4.35E-07	2.32E-05	6.70E-03	0.248
Macroglossia	**ARS-USDA-AGIL-chr4-114395607-000108***	113596650	4	95	A	G	6.86E-04	0.31	0.09	0.41	6.42E-06	1.58E-04	4.05E-02	0.200
Macroglossia	ARS-USDA-AGIL-chr7-18201332-000761	16970401	7	95	C	A	1.94E-01	0.13	0.30	0.05	1.56E-06	5.75E-05	1.43E-02	0.227
Macroglossia	**ARS-USDA-AGIL-chr10-25594159-000176***	25539231	10	95	G	A	3.91E-02	0.47	0.23	0.59	4.07E-06	1.14E-04	2.94E-02	0.209
Macroglossia	**ARS-USDA-AGIL-chr11-36809347-000009***	36957396	11	95	A	C	1.95E-03	0.37	0.13	0.49	7.14E-07	3.30E-05	9.01E-03	0.239
Macroglossia	ARS-BFGL-NGS-15423	72258249	16	95	G	A	4.35E-05	0.22	0.44	0.11	2.99E-07	1.78E-05	6.03E-03	0.252
Macroglossia	BovineHD1600007856	27494192	16	95	A	G	4.45E-01	0.28	0.52	0.16	2.17E-07	1.42E-05	6.03E-03	0.261
Macroglossia	BovineHD4100012725	72266300	16	95	A	G	1.09E-03	0.19	0.39	0.10	1.18E-06	4.71E-05	1.19E-02	0.230
Macroglossia	ARS-USDA-AGIL-chr17-6999864-000318	7013884	17	95	G	A	4.14E-04	0.45	0.20	0.58	8.46E-07	3.73E-05	9.49E-03	0.235
Macroglossia	**ARS-USDA-AGIL-chr22-32285822-000477***	32169050	22	95	A	G	1.49E-03	0.25	0.47	0.13	4.65E-07	2.43E-05	6.70E-03	0.246
Macroglossia	**ARS-USDA-AGIL-chr24-25995108-000530***	25684356	24	95	A	C	1.69E-05	0.31	0.03	0.44	5.06E-09	9.83E-07	5.10E-04	0.319
Macroglossia	BovineHD2600011259	40460199	26	95	A	G	3.54E-02	0.43	0.70	0.29	7.21E-08	6.47E-06	3.64E-03	0.278
Macroglossia	BovineHD2600011282	40517972	26	95	G	A	2.17E-01	0.46	0.72	0.33	2.70E-07	1.65E-05	6.03E-03	0.257
Macroglossia	BovineHD2600014129	48680201	26	95	G	A	8.05E-01	0.29	0.52	0.18	1.94E-06	6.73E-05	1.63E-02	0.224
Macroglossia	BovineHD4100017966	40441709	26	95	A	G	5.02E-01	0.36	0.58	0.25	6.39E-06	1.57E-04	4.05E-02	0.203

*** In common between the two patologies**

* shared by the two syndromes

Table S4: Quadratic Distance between animals affected and healthy control for each comparison.

Disease Status	Ar	Ma	He
Arthrogryposis (Ar)	-	7.83***	7.99***
Macroglossia (Ma)			14.14***
Healthy control (He)			-

***significance of Mahalanobis distance between compared groups (p-values <0.001)
